# Future Perspectives for Hand Transplant in Iran

**Published:** 2011-11-01

**Authors:** M. J. Fatemi, M. Masoumi, E. Esfandiari

**Affiliations:** 1 *Department of Plastic Surgery, Tehran University of Medical Sciences, Tehran, Iran, *; 2 *Traumatic Veterans Management Center, Foundation of Martyrs and Veterans Affair, Tehran, Iran*

**Keywords:** Hand, Transplantation, Allotransplant, Upper limb amputation

## Abstract

Hand transplant program is a communion of physicians and researchers during the current decade. 72 hands and digits were transplanted in 53 patients over the past 13 years. Unlike a solid organ transplant, hand transplantation involves various tissues, so it is called “composite tissue allotransplantation.” This article discusses the plans for performing the first hand transplant in Iran.

## INTRODUCTION

composite tissue allotransplantations include skin, subcutaneous tissue, muscle, bone, cartilage, tendon, nerve and blood vessels. Unlike solid organ transplant (*i.e.*, kidney, liver and heart) which consists of homogenous parenchymal tissue, composite allografts are homogenous tissues with different degrees of antigens that can cause strong immune response [1]. Composite tissue allotransplant falls under the reconstructive surgery category, has potential of microvascular surgery principles with combination of organ transplantation [2-7]. Solid organ transplantation is extensively accepted in the medical community as the best treatment for end-stage conditions. However, different opinions exist towards composite tissue allotransplantation in general and hand transplant in particular [8-12]. 

Hand has a very important role in body image and sense of identity, work, relationships, *etc* [13-15]. The first hand transplantation was done in France in 1998 by an international team of surgeons [16]. The results of this first operation reached to surgical technique feasibility, immunosuppressive efficacy and the importance of patient cooperation and rehabilitation program.

Immunosuppressive treatment for acute rejection of organ allografts has been effective [17]. By advent of novel immunosuppressive therapies which include non-steroidal medications, compliance to long-term treatments is reduced. The only rejection of hand transplant was reported two years after the first procedure and the cause was the cessation of immunosuppressive treatment.

According to a survey in 2005 on veterans with bilateral upper limb loss in Iran, more than two-thirds of amputees had below elbow amputation and just a few of them were using prosthesis. It seems that considering improvement in hand transplant around the world and the problems veterans are faced, this procedure would be helpful for functional recovery from upper limb loss. Based on current reviews, foundation of Martyrs and Veterans affair with cooperation of Iran medical Universities would like to do the first hand transplantation in Iran. This article discusses the plans for performing the first hand transplant in Iran.

Hand Transplantation around the World 

The International Registry on Hand and Composite Tissue Transplantation (IRHCTT) was established in May 2002, to create cooperation among all teams performing hand transplantation [18-20].

Globally, 72 hands transplants (30 unilateral and 42 bilateral hand transplantations) and two digits in 53 patients were reported to IRHCTT from September 1998 to 2011. The last case was a double hand transplantation in Melbourne, Australia.

The hand transplant program is a communion of physicians and researchers during the current decade. Literature review reflects satisfaction of all transplantations except for Chinese ones [5]. The only reason reported was disassociation of patients, noncompliance or unavailability of immunosuppressive drugs.

Ethics Process

All potential risks and complications are explained to patients. Those include the need for additional surgery, graft rejection, complications of the immunosuppression, metabolic abnormalities, death, *etc*.

Ideal Candidates for Hand Transplantation

Good candidates for hand transplantation are patients who are highly motivated and had clean-out bilateral mid- or distal-forearm amputations [17]. For early post-operative active mobilization of the wrist and fingers, preservation of extrinsic muscles is required.

Pre-operative Evaluation

Pre-operative hand transplant evaluation consists of two main aspects [21]: Physical evaluation and psychological evaluation. History taking, physical examination, angiogram of both forearms for assessing vessel status, MRI, ultrasound, and EMG are covered in physical evaluation. A detailed laboratory work, blood typing, human leukocyte antigen (HLA) typing, human immunodeficiency virus screening, *etc*, are included in a detailed evaluation [21].

In psychological evaluation, various aspects including emotional evaluation, the capacity of recipient to make decision correctly, Rorschach/thematic apperception testing, evaluation of patient’s family support, financial status, *etc*, are evaluated [21]. The most important aspect in pre-operative evaluation is recipient’s medical compliance history. 

Donor Selection

Donors were selected based on blood type, race, gender, size and skin color matching. HLA matching, negative lymphocytotoxic cross-matching, B and T cell matching are performed.

Surgical Procedure

The operative technique for hand transplantation has been reported in several studies [18, 21]. In summary the sequence of procedure for tissue repair is bony fixation, arterial revascularization, vein repair, tendon repair, and nerve repair. Heparin administered for 48 hours post-operatively is an important issue for all patients. 

Immunosuppressions

The first hand transplantation in Ecuador in 1964 was unsuccessful for lack of enough immunosuppression therapies in that time [22]. Since 1998 when the first successful hand transplant was done [19], a wide range of immunosuppression therapies have been employed; treatments consisted of induction therapies, and appropriate drugs for maintenance therapy, control of rejection, and prophylaxis for opportunistic infections.

Physical therapy and Rehabilitation

Physiotherapy is a necessary step after hand transplantation, preferably to be started on the first post-operative day. Passive movements are started first. Active movement exercises are used as soon as possible. In the absence of exercises, hand should be supported in a thermoplastic splint in intrinsic plus position. Sensory re-education and occupational therapy are indicated in later phases. The movements and exercises should be intense and for a long time [17].

Results Evaluation

As it would be difficult to analyze transplantation functional results, a grading system was proposed by Lanzetta, *et al* [2]. The hand transplantation score system (HTSS) evaluates six aspects with different weight for a total of 100 points: Appearance (15 points), sensibility (20 points), movement (20 points), psychological and social acceptance (15 points), daily activities and work status (15 points), and patient satisfaction and general well-being (15 points). A total score of 81 to 100 points is considered an “excellent outcome,” a score of 61 to 80 is “good,” 31 to 60 “fair,” and a score of 0 to 30 is considered “poor.”

## FUTURE PERSPECTIVES FOR HAND TRANSPLANT IN IRAN

Hand transplantation with a high success rate and satisfactory functional outcome would be efficient for persons with upper limb loss, specifically for those with bilateral amputations [23]. 

With improvement in immunosuppressants, hand transplantation became a reality [5]. For avoidance of rejection and achieving good functional results, compliance of the recipients to immunosuppressive therapies and rehabilitation program is essential.

To perform the first hand transplantation in Iran, we had to make it clear if the procedure is legal from the Islamic point of view and thus we asked Apostle Makarem Shirazi about the legality of hand transplant in Islam. Based on his verdict, if the donor is brain-death, the procedure is permitted.

Another step was orchestrating a team in Sasan Hospital, Tehran, consisting of an orthopedic surgeon, a hand surgeon, physical and rehabilitation physicians, a physiotherapist, a dermatologist, a pharmacologist, an immunologist, a pathologist, a psychologist, an occupational therapist and an orthotist.

Since the list of the first hand transplantation candidates was ready at this point, the only work to be done is evaluation of volunteers by a psychologist and an immunologist.

The protocol of pre-operative evaluation consists of a psychological evaluation, physical and immunological examinations. We think that no special equipment is needed, and all the requirements for the surgery are existed. Information on post-operative rehabilitation program has been assembled. Nonetheless, contacting previous hand transplant rehabilitation teams to make the best decision and evaluation is necessary.
